# Predator‐guild‐specific parental responses mitigate higher predation risk on ground nests close to forest patches in a mosaic landscape

**DOI:** 10.1111/1365-2656.70278

**Published:** 2026-05-18

**Authors:** Guillaume Dillenseger, Justine Delautre, Ankitha Jayanth, Zora Marchal, Petr Veselý, Vojtěch Kubelka

**Affiliations:** ^1^ Department of Zoology, Faculty of Science University of South Bohemia České Budějovice Czech Republic; ^2^ Faculté Des Sciences Université de Montpellier Montpellier France; ^3^ Biological and Life Sciences Department Ahmedabad University Gujarat India; ^4^ Faculté Des Sciences de la Vie Université de Strasbourg Strasbourg France; ^5^ Centre for Polar Ecology, Faculty of Science University of South Bohemia České Budějovice Czech Republic

**Keywords:** agricultural landscape, anti‐predatory behaviour, birds, edge effect, landscape of fear, lapwings, nest defence, predator recognition

## Abstract

1. The risk of nest predation differs across habitats and increases with proximity to habitat edges. In agricultural mosaic landscapes, the breeding success of ground‐nesting birds can be negatively impacted by the presence of adjacent forest patches, which act as a source of predation. However, we still have limited knowledge on how birds nesting in such habitats cope with variable predation risk.
2. In this study, we investigated predation rates of experimental ground nests and Northern Lapwing nests (*Vanellus vanellus*), in relation to the nearest patch. Furthermore, we tested the aggressive responses displayed by nesting pairs towards stuffed nest predators of different guilds at different distances from the forest.
3. Predation rate on experimental nests increased with proximity and area of the closest forest patch. In contrast, predation rate on lapwing nests was not affected by the presence of forest, suggesting effective nest defence performed by parents to protect their clutch. Pairs displayed more aggressive behaviours against predators compared with a control wood log. Additionally, responses to specific predators varied with forest distance. Lapwings appeared more aggressive towards a bird when closer to forests, but were more aggressive towards a mammal further from forests.
4. This study highlights the importance of fine‐scale differences in anti‐predatory behaviours to compensate for local variations in predation risk, in heterogeneous agricultural landscapes.

## Graphical abstract

Predation increases close to habitat edges, but how animals cope with local predation variations remained poorly studied. In a mosaic landscape, lapwings were compensating for increased nest predation close to forests. They acted more aggressively towards an avian predator when nesting close to trees, but comparatively tamer against a mammal.

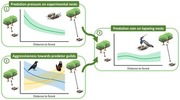

## 1 INTRODUCTION

Predation represents the primary cause of nest loss among birds (Martin, 1993a; Ricklefs, 1969). Several predatory guilds are involved in nest predation attempts, mammals and birds being the most common in temperate regions (Barton et al., 2026). Ground‐nesting birds appear vulnerable to both terrestrial and aerial predators, due to high accessibility of the clutch (Martin, 1993b). However, the risk of predation is spatially heterogeneous, with some sites exposed to higher predation pressure. Nest predation tends to be stronger closer to habitat edges, notably for ground nests (Batáry & Báldi, 2004; Hartley & Hunter, 1998). Higher predation rates at habitat edges result from predatory guilds exploiting and using them as corridors (Svobodová et al., 2011). Over the last century, increased anthropogenic activities and agricultural intensification have amplified habitat fragmentation, and consequently edge effects, hence negatively impacting bird species breeding in agricultural mosaic landscapes (Burger et al., 1994; Fletcher Jr, 2005; Franks et al., 2017; Porensky & Young, 2013).

As clutch predation acts as a strong selective pressure on breeding individuals to maintain high reproductive success, most avian species have evolved an array of anti‐predatory strategies (Caro, 2005). When it comes to defending their offspring, parents are faced with a trade‐off between protecting their clutch, hence ensuring a successful breeding attempt, or risking being injured or killed (Lima, 2009; Montgomerie & Weatherhead, 1988). Anti‐predatory strategies can successfully prevent depredation, but different strategies may be more efficient against specific predatory guilds, such as ground displays being efficient for distracting mammals (Byrkjedal, 1987; Gochfeld, 1984). Nest site selection may also substantially affect predation risk (Mainwaring et al., 2015). Thus, breeding individuals are expected to select sites to reduce the risk of depredation (Rosenzweig, 1981). For instance, several bird populations avoid building nests close to forest edges in mosaic landscapes (Bertholdt et al., 2017; Pálsdóttir et al., 2022), associated with a lower survival closer to trees (Storch et al., 2005). The choice of nest site may be driven by personal experience of previous predation attempts (Chalfoun & Martin, 2010), and indirect assessment of predation risk through environmental cues (Tolvanen et al., 2018). Other parameters could influence this decision; nesting site selection could be the result of a trade‐off to maximise abiotic conditions during the incubation period (Marzluff, 1988; Tieleman et al., 2008). Furthermore, local predation risk could vary between predator guilds while remaining constant over the whole environment, which limits the optimisation of nest site selection (Kleindorfer et al., 2005; Martin, 1993a).

Ultimately, individuals vary in their responses towards predators between different nest locations. Birds can act more boldly when nesting in sites with higher predation risks (Clermont et al., 2021; Seltmann et al., 2014). Specific responses towards a given predator appear stronger in locations where it is commonly encountered (Liu et al., 2025; Sandoval & Wilson, 2012). These observations fit within the concept of landscape‐of‐fear (Gaynor et al., 2019). However, we still lack evidence on how parental responses differ between predatory guilds in landscapes with heterogeneous predation risk. In mosaic agricultural landscapes, which incorporate patches of other habitats, nest predation often increases closer to forest edges, as woodland patches serve as habitat and corridors for predators (Andren, 1992; Červinka et al., 2013). Mammalian predators are often identified as occurring close to forest patches and being responsible for this edge effect (Červinka et al., 2011; Kaasiku et al., 2022; Storch et al., 2005). However, the specific consequences of higher mammal occurrence for birds in agricultural landscapes have not been fully addressed, especially regarding behavioural adjustments.

Studies on nest predation often rely on experimental design, using commercially bought eggs (Burger et al., 1994; McKinnon et al., 2010). Experimental nests without parents defending them correspond to naïve models that measure the predation pressure experienced by individuals (Freeman et al., 2020). However, the predation pressure on experimental nests is poorly correlated with real nest predation rates, as parental defence can successfully deter predation attempts or select safer nest sites (Freeman et al., 2020; Jayanth, 2024). Additional methodological biases could affect predation rates on experimental nests. Human scent deposited during installation could attract rodents but deter large mammals from approaching (Rangen et al., 2000), while constant solar exposure affects shell colouration, which could make nests more visible to avian predators (Cembrano et al., 2021). Nevertheless, experimental nest designs remain a simple standardised procedure to evaluate the local predation pressure experienced by wild populations, but need to be distinguished from real nest predation measures, which inform about the local adaptations of prey.

In this study, (A) we measured the effect of forest distance and area on predation pressure by monitoring the fate of experimental nests. (B) We compared it to the predation rate of lapwing nests, which informed us if populations were adapted to local risk and counterbalanced variations in predation. (C) We then assessed if behavioural responses of individual parents varied depending on their proximity to the closest forest patch to determine if birds were able to cope with fine‐scale variations in predation risk. For this, we tested the responses of pairs breeding at different distances from forest patches to stuffed nest predators of different guilds.

We hypothesised that (A) the predation rate of experimental nests increased with forest proximity, because of higher predator occurrence closer to edges, and with larger forest patches, potentially inhabited by higher predator densities or diversity. However, (B) we expected that predation on lapwing nests would not be affected by forest distance nor area, as parents might successfully protect their nests from predation attempts. (C) We hypothesised that breeding birds would be more aggressive when nesting closer to forest edges and larger forest patches, as these individuals might encounter predators more often. When comparing their behaviours towards specific guilds, we expected birds to be more aggressive towards mammalian and avian predators, compared with a control stimulus, as the former represent a potential risk for clutch survival. Additionally, we expected individuals to act more aggressively towards a mammal when nesting closer to the forest and near larger patches, as they might encounter mammals more often. Comparatively, this effect could either be less pronounced for avian predators, as birds can be encountered everywhere, or still stronger close to the trees, as predatory birds may benefit from perches while hunting.

## 2 MATERIALS AND METHODS

### 2.1 Study area and species

**FIGURE 1:**
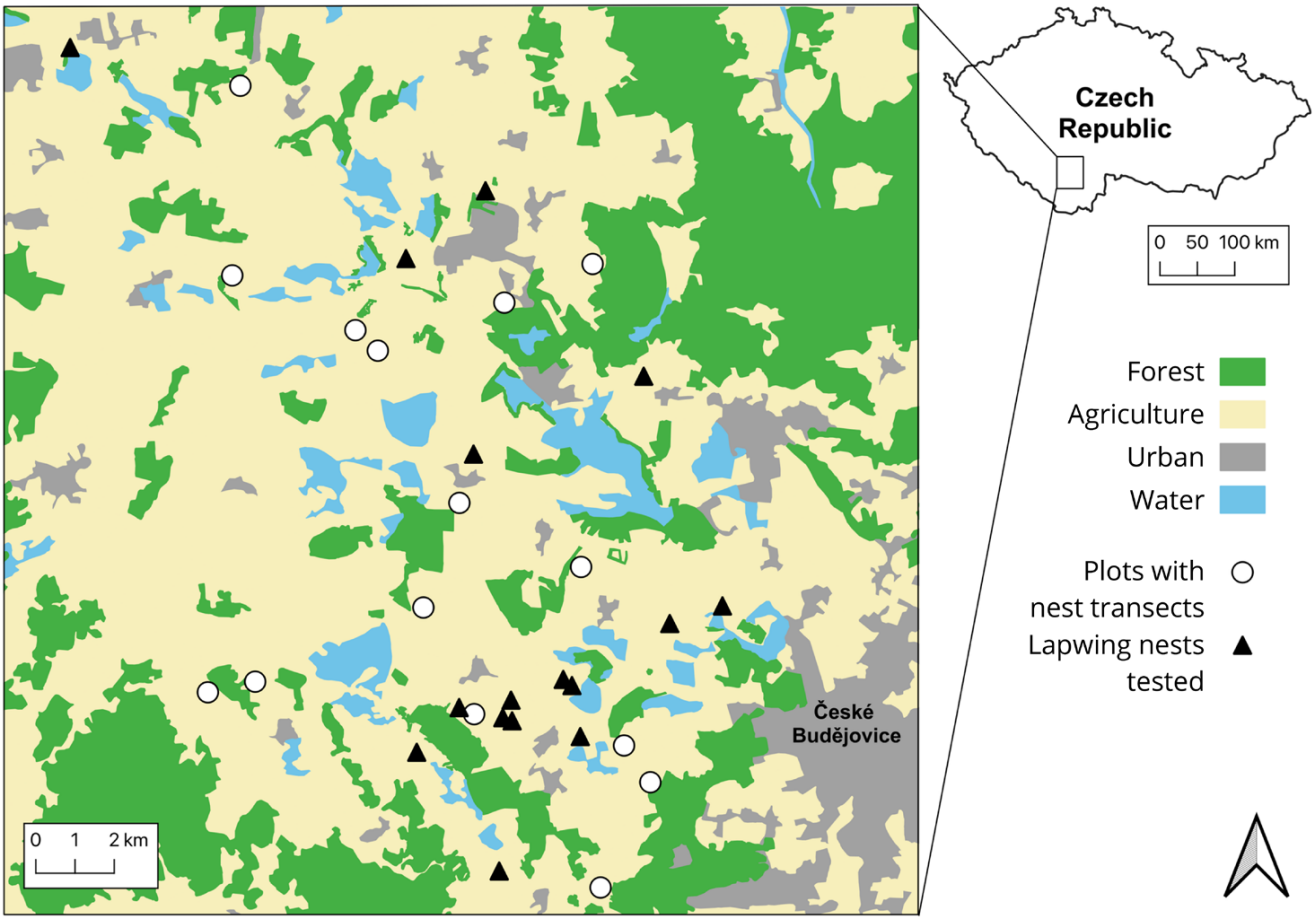
Map of the study area within Czechia during 2024–2025 breeding seasons, with landscape composition and location of agricultural plots with experimental nest transects (*n* = 15) and real lapwing nests presented with dummies (*n* = 16). Made with QGIS.

This study was performed around České Budějovice, in the South Bohemia region, Czechia (49.0° N, 14.3° E; Figure 1), during the 2024 and 2025 breeding seasons. The landscape is dominated by agricultural fields, interspersed with forest patches of variable sizes and artificial ponds. Several ground‐nesting bird species commonly breed in grasslands and agricultural patches. We divided the area into ‘localities’, which refer to continua of agricultural and grassland plots, delimited by other habitats or roads.

We focused on breeding pairs of Northern Lapwing (*Vanellus vanellus*) (hereafter lapwing). The lapwing is a medium‐sized shorebird (family Charadriidae), associated with open habitats, which lays a clutch of 3–4 eggs on the ground and incubates for 27 days (Shrubb, 2010). Nest care can be provided by both parents or just one. Lapwings often breed in loose colonies, and perform diverse anti‐predator behaviours during breeding. These characteristics make them a great model to observe parental defence and aggressiveness during nesting season (Elliot, 1985; Królikowska et al., 2016). The lapwing population around České Budějovice has been monitored since 1988, and annually since 2010 (Jayanth, 2024; Kubelka et al., 2018). The work was permitted by the Ministry of Agriculture of Czechia (n°43,873/2019‐MZE‐18134).

### 2.2 Predation rate on experimental nests

To assess predation pressure, we measured predation risk at different distances from forest patches using an experimental nest design. For this, we created open scrapes resembling lapwing nests by forming a small depression on the ground and placing three quail eggs (*Coturnix japonica*) in each, with the pointed end directed towards the centre of the nest. Quail eggs closely resemble the colouration of shorebird eggs in visible and ultraviolet spectra (Cembrano et al., 2021), and are recommended when predator communities are dominated by large species (Rangen et al., 2000), which were assessed previously (Baraldi, 2025). A total of 100 nests (*n* = 60 in 2024; *n* = 40 in 2025) were installed in 15 agricultural plots, along 28 transects perpendicular to the edges of a priori selected forest patches, and placed at standardised distances from the edge: 50, 100, 300 (*n* = 28 for each) and 500 m (*n* = 16). Nests were placed around mid‐April of both years to synchronise with the local breeding peak (Jayanth, 2024), all within a period of 10 days (2024: April 19–29; 2025: April 23–28), thus avoiding temporal changes in nest predation rates throughout the breeding season. When placing each nest, we recorded GPS coordinates, and placed two inconspicuous wooden popsicle sticks at 1 and 2 m from the nest to relocate it later. We placed the experimental nests in fields that were adjacent and similar to the ones where we monitored the lapwing nests, but avoided placing them closer than 100 m to known lapwing nests as the behaviour of parents could reduce predation rates on neighbouring nests. Habitat type was recorded during installation: corn (*n* = 31), legume (pea or clover) (*n* = 10), spring (*n* = 8) or winter crops (*n* = 13), ploughed/harrowed (*n* = 12), stubble fields (*n* = 8) or grasslands (*n* = 18).

Forest patches were selected a priori such that the transects consisted of a continuum of agricultural patches, not interrupted by roads or other habitats. Experimental nests were more than 500 m away from any other forest patches, apart from the one they were associated with. Following both restrictions, a limited number of patches allowed nests to be placed at 500 m, explaining the lower sample size. We ensured a gap of at least 100 m between two transects if they shared the same forest patch. A forest patch was defined as a group of trees with overlapping canopy, covering at least 1 ha. Forest patch sizes were measured (±0.1 ha) with QGIS v.3.40.5 (QGIS Geographic Information System, 2024).

We visited the nests at day +1, +3, +5 and then every 5 days after installation, until day +25, a period similar to the lapwing incubation length of 27 days (Shrubb, 2010). We visited them more frequently at the onset, as we expected predation events to happen quickly after installation. We considered a nest predated if an egg was either missing or damaged. We revisited the nest until all three eggs were missing or consumed, which we considered predated, or until it survived to day +25, which we considered successful. We did not account for partial predation, as a nest with only one egg surviving would be considered a successful attempt for lapwings. Experimental nests that were destroyed by agricultural machinery (*n* = 11) were removed from the analysis. Total predation rates were calculated as the proportion of predated nests out of the total of nests.

### 2.3 Predation rate on lapwing nests

We compared the standardised predation risk on experimental nests with the actual predation rates of encountered lapwing nests.

At the beginning of each breeding season, we located lapwing nests by observing incubating parents in suitable habitats. We recorded GPS coordinates of the nests (Garmin eTrex 10) and placed bamboo poles 10 m from the nest along crop lines to protect them from agricultural machinery. This method does not increase nest predation risk (Zámečník et al., 2018). We measured the incubation stage as the number of days and estimated the hatching date of each clutch with the flotation method (van Päässen et al., 1984). We revisited each nest three times until hatching to assess its fate. We assumed that eggs successfully hatched based on the presence of chicks at the nest or tiny eggshell fragments at the bottom of the scrape (Green et al., 1987). We considered a nest successful when at least one egg hatched. Missing fragments or a damaged clutch indicated a predation event. We were able to assess the fate for 317 nests (*n* = 218 in 2024; *n* = 99 in 2025). Habitat type was recorded: corn (*n* = 81), legume (*n* = 2), rapeseed (*n* = 3), spring (*n* = 11) or winter crops (*n* = 40), ploughed/harrowed (*n* = 144), stubble fields (*n* = 15) or grasslands (*n* = 21).

To identify the forest patch closest to each nest, we calculated the shortest distance to the nearest forest patch (±1 m) based on GPS coordinates of the nests and all forest patches identified from CORINE Land Cover 2018 and Google Maps orthophotos using QGIS (46–1596 m; mean = 518 m). Lapwing nests could be further than 500 m from any forest because we ignored habitat continuity for this measurement. Area of the closest forest patch was measured as for experimental nests (1.3–1179.8 ha; mean = 281.3 ha). Daily predation rates were calculated using the Mayfield method, as described in Jayanth (2024).

### 2.4 Behavioural testing

To measure parental aggressiveness of lapwings with regards to forest distance, we exposed nesting pairs to nest predator dummies. We selected 16 monitored nests, visited only once before for our experimental design. On the day of the experiment, nests reached at least one‐third of incubation (12–26 days; mean = 18.7 days). We avoided the first part of incubation to reduce the risk of nest abandonment, as parental investment increases with incubation advancement (Królikowska et al., 2016). None of the tested birds were ringed or tagged, as there could be effects of previous manipulations on bird reactions (Oñate‐Casado et al., 2021). Tested lapwing nests were sufficiently distant from each other, so that we could not see two of them from a point on the ground, and birds were unlikely to have witnessed the design before being tested (250–10,124 m; mean = 2614 m).

Four lapwing nests were exposed to two possible stimuli during the 2024 breeding season: a stuffed European Polecat (*Mustela putorius*) as mammalian predator, and a wood log as control. In 2025, 12 nests were tested using the same protocol, with the addition of an avian predator: a stuffed Carrion Crow (*Corvus corone*). Both species are common predators of lapwing nests (Teunissen et al., 2008) and were present in our field locations (Zámečník et al., 2018). Dummies were prepared in natural positions and had similar heights once placed: the crow was perched with folded wings; the polecat was standing upright on hind legs; the log was placed vertically. We opted for a mustelid dummy as the size of the stimulus can affect bird responses (Fišer et al., 2024), and selected two stimuli of similar size. Behavioural experiments were carried out outside of precipitation events.

One dummy was brought to the nest at a time, covered with a cloth to limit association with human disturbance and avoid premature responses of parents, and placed 2 m away from the nest, facing the nest, perpendicular to the observer's direction. The cloth was removed upon departure. When a human entered a field, birds would usually fly away or circle the experimenter until they left. Reactions of the nesting birds were recorded with a camera (several models, zoom ≥50×), placed at a distance from which the birds kept incubating, unalarmed, under normal conditions (111–457 m; mean = 273 m). As soon as a parent was located, we started to record 15 min of interactions between the bird(s) and the dummy. As birds were unmarked, we could not ascertain paternity in most cases unless the birds came back to incubate. Hence, we considered the two closest and most aggressive individuals to be the parents. We always observed a maximum of two adults of different sexes displaying aggressive behaviours towards the dummies; thus, we considered our approach to enable confident identification of the parents. Lapwings may present sexual dichromatism, but not all pairs could be visually sexed, hence we could not identify individuals with certainty between different trials, nor use individual identity as random effect. Often only one parent came back to the nest during the experiment. After 15 min of interactions, the dummy was removed, and the birds were given at least 2 h before being presented with the next dummy.

Stimuli were presented in a random order, preferably on the same day to avoid temporal bias and risk of predation between experiments. Out of the 16 tested nests, none were abandoned following the experiments. After the hatching date, we assessed whether the lapwing nest hatched successfully. For the 12 nests for which we were able to confirm the fate, 100% successfully hatched.

### 2.5 Behavioural coding

Videos were analysed using Boris v.9.1.1 (Friard & Gamba, 2016), by a single observer. We recorded all behaviours displayed by the parents of the focal nest (Supporting Information S1) within the period of 15 min that started when one parent came back and was spotted by the observer in the field. Reactions to humans were ignored in the analysis. We were able to analyse 42 trials (*n* = 15 with log; *n* = 12 with crow; *n* = 15 with polecat). This represented more than 11 h of observations. We always considered two parents, except for a single uniparental nest. In the case where only one parent interacted during the experiment, the second adult was coded as ‘Away’. We coded all behaviours as mutually exclusive state events, resulting in individuals performing one behaviour at a time.

For each behaviour, we attributed an aggressiveness score ranging from 1 (non‐aggressive behaviours) to 4 (most aggressive behaviour), and 0 for individuals that remained away, which we considered as a lack of aggressive response (Table 1).

**TABLE 1:** Semiquantitative aggressiveness score attributed for each behaviour displayed by breeding lapwings during dummy presentation experiments.

Score	Behaviours
0	Away
1	Ignoring/incubating/flying
2	Vigilance far/vigilance close/
3	Broken‐wing display/circling
4	Attacking

*Note*: The complete ethogram with definitions is available in Supporting Information S1.

### 2.6 Statistical analyses

Statistical analyses were performed in R v.4.4.2 (R Core Team, 2024). We performed experimental and lapwing nest survival analyses using cox hazard ratio models of the ‘coxme’ package v.2.2–22 (Therneau, 2022). This procedure estimated how variables affected the instantaneous risk of predation, and allowed us to account for the random effect of the localities as a categorical variable. We created exposure histories, or time‐to‐event data, for each nest with exposure time in days and event being the fate of the nest: ‘1’ if predated; ‘0’ if survived or hatched. For both experimental and lapwing nests, we tested the time‐to‐event data with distance and area of the closest forest patch, both as continuous, and habitat types as fixed effects, and locality as a random effect, to account for spatial similarities. Treating distance as continuous reduced the potential influence of unbalanced sampling of nests at 500 m. The results of the models provided cox hazard ratios for each predictor, which represent relative differences in predation risk. Hazard ratios >1 indicated a positive correlation between the predictor and the predation risk, whereas ratios <1 indicated a negative correlation. For ‘Habitat’, the reference level was the first in alphabetical order.

To test for variations in lapwing aggressiveness according to forest characteristics, we accounted for the highest aggressiveness score displayed by each individual during a session (Table 1). We tested the individual's highest score as a continuous variable in a linear mixed‐effect model (LMM) with nest identity as a random variable, using package ‘lmerTest’ v.3.1–3 (Kuznetsova et al., 2017). We opted to treat the aggressiveness score as continuous rather than ordinal, as we expected linear relationships between the response variable and predictors, and the data violated the proportional odds assumption for the use of ordinal models (Brant test: *p* = 0.027) (Robitzsch, 2020; Ugba, 2022). We centred continuous variables to highlight the main effects of the dummy, and we tested the effect of the distance and area of closest forest patch, dummy and the interactions between dummy and forest variables in a single model.

To control for the effects of incubation stage and interactions between the dummy and presentation order and year, we ran likelihood ratio tests (LRT) and evaluated the change in model fit with Chi‐square *p*‐values using function ‘add1’. We assessed the normality of residuals graphically for all models.

## 3 RESULTS

### 3.1 Predation rate on experimental nests

**FIGURE 2:**
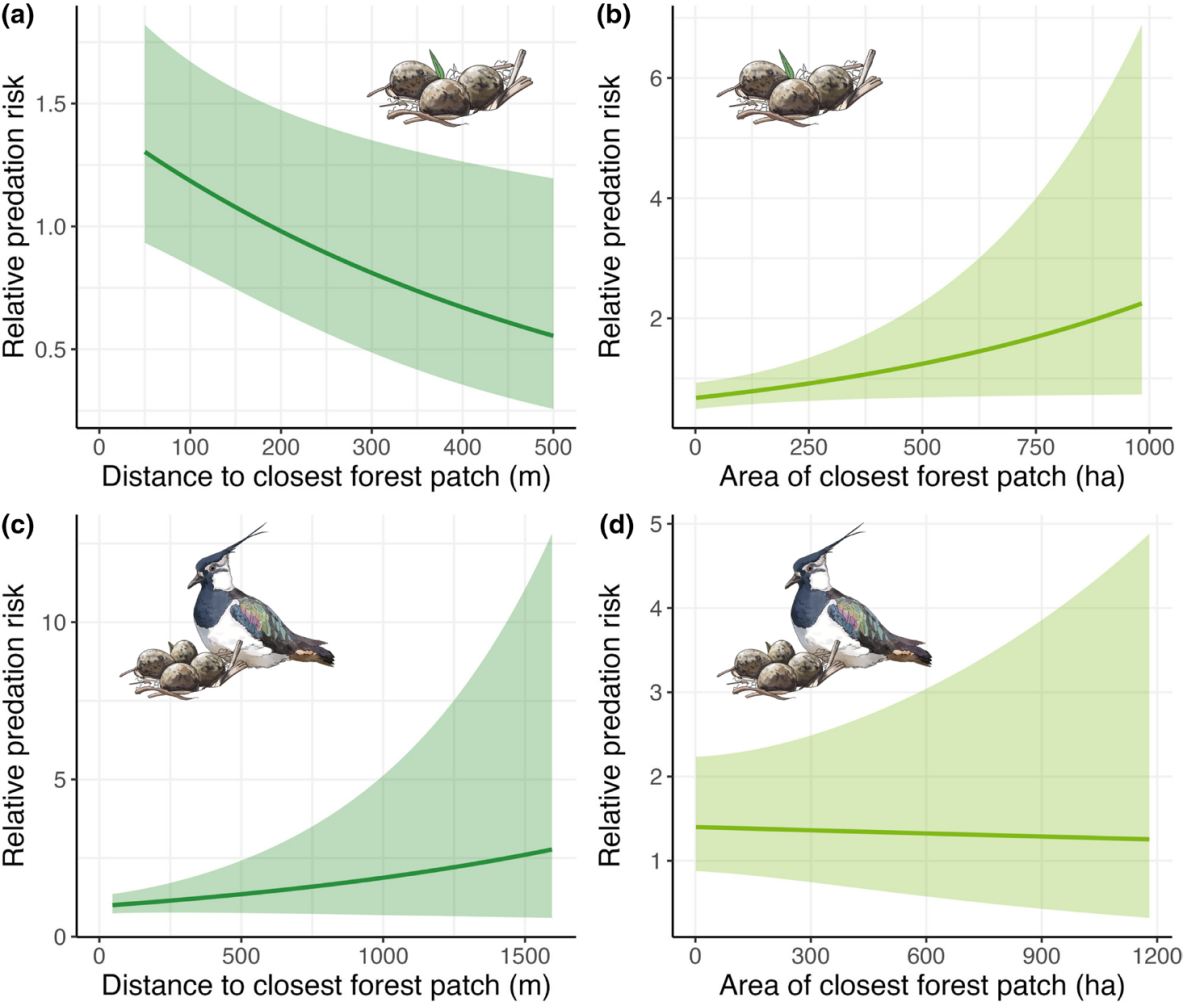
Relative predation risk for experimental nests (*n* = 89) (a) according to the distance to the closest forest patch (in metres); (b) and the area of the closest forest patch (in hectares); and for lapwing nests (*n* = 317) (c) according to forest distance; (d) and forest area, predicted with the cox hazard ratio model; different scales; shaded areas represent 95% confidence interval.

The total predation rate of experimental nests reached 96.63% (*n* = 89) and varied with distance to forest: 96.00% of nests at 50 m were predated (*n* = 25); 100.00% at 100 m (*n* = 24); 88.46% at 300 m (*n* = 26); and 85.71% at 500 m (*n* = 14). When modelled using hazard ratios, relative predation risk decreased with distance to the closest forest patch, but increased with its area (Figure 2a,b). Habitat type explained differences in predation risk, with slightly lower risk in corn compared with grasslands, spring crop, ploughed and stubble fields (Table 2).

**TABLE 2:** Results of cox hazard ratio models for experimental nests placed at 50, 100, 300 or 500 m from selected forest patches along 28 transects (locality random effects: Variance = 0.3337; standard deviation = 0.5776), and for monitored lapwing nests (locality random effects; variance = 0.2872; standard deviation = 0.5359) with 95% confidence intervals.

Datasets	Predictors	Estimates	95% CI	p
Experimental nests (*n* = 89)	Distance to closest forest patch	0.9981	0.9966–0.9996	**0.015**
	Area of closest forest patch	1.0012	1.0001–1.0024	**0.039**
	Habitat: grassland	3.1381	1.1615–8.4787	**0.024**
	Habitat: legume	2.5906	0.7891–8.5043	0.117
	Habitat: ploughed	12.2573	3.0387–49.4423	**<0.001**
	Habitat: spring crop	4.2092	1.1855–14.9449	**0.026**
	Habitat: stubble	18.3933	4.7220–71.6465	**<0.001**
	Habitat: winter crop	1.5609	0.6703–3.6347	0.302
Lapwing nests (*n* = 317)	Distance to closest forest patch	1.0007	0.9997–1.0016	0.157
	Area of closest forest patch	0.9999	0.9989–1.0009	0.856
	Habitat: grassland	1.0237	0.3049–3.4372	0.970
	Habitat: legume	5.6128	0.5431–58.0044	0.148
	Habitat: rapeseed	0.0000	0.0000 – Inf	0.999
	Habitat: ploughed	1.0024	0.5300–1.8959	0.994
	Habitat: spring crop	0.3336	0.0406–2.7423	0.307
	Habitat: stubble	3.6712	1.2679–10.6295	**0.017**
	Habitat: winter crop	1.6559	0.7009–3.9118	0.250

*Note*: ‘Habitat’ categories are compared with reference level ‘corn’; bold values highlight significance levels: *P* ≤ 0.05.

### 3.2 Predation rate on lapwing nests

Total predation rate for lapwing nests reached 24.54%, which corresponded to a daily predation rate of 1.00% (*n* = 317). When predicted with hazard ratios, forest characteristics did not influence predation risk (Figure 2c,d). Predation risk appeared higher in stubble fields compared with corn fields (Table 2).

### 3.3 Behavioural responses of breeding birds

**FIGURE 3:**
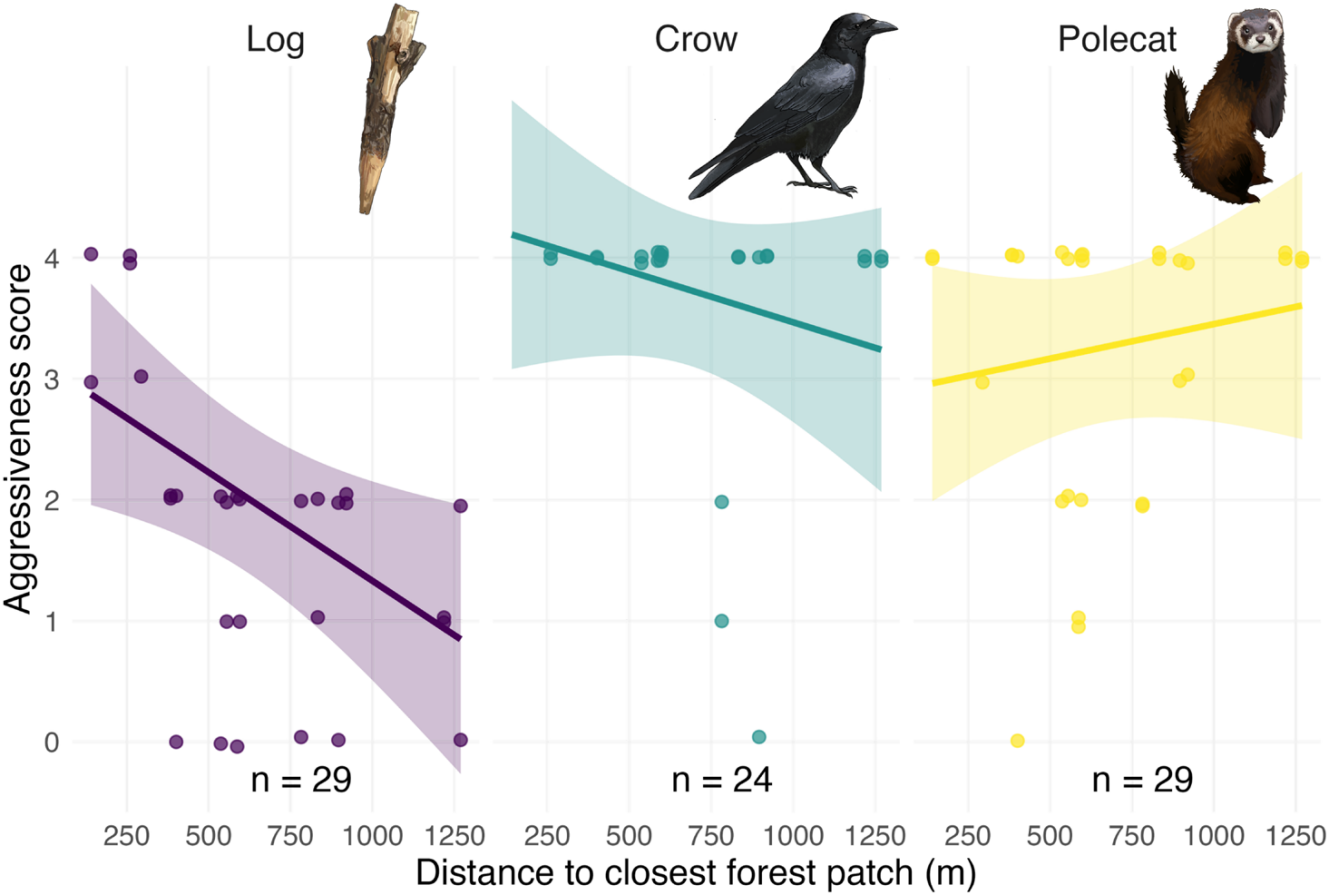
Predicted aggressiveness scores of breeding lapwing individuals from 16 nests according to the distance between their nest and the closest forest patch (in metres) when exposed to stuffed nest predators; coloured dots represent individual scores and are jittered to highlight overlapping points; sample sizes indicate number of individuals.

Breeding lapwings acted more aggressively at closer distances to forest patches, but not with area of the forest (Table 3). Lapwings displayed higher scores against both the crow and the polecat, compared with the log. Interactions between polecat dummy and forest distance significantly differed from the wood log control. Thus, nesting lapwings acted more aggressively towards the polecat when their nests were further from forest, unlike for the crow and the log (Figure 3; Table 3). Addition of neither nest age (LRT_1_ = 0.002; *p* = 0.961), nor interaction between presentation order and dummy (LRT_6_ = 8.682; *p* = 0.192), nor interaction between year and dummy (LRT_2_ = 3.771; *p* = 0.152) improved the model.

**TABLE 3:** Results of LMM models with centred continuous variables for aggressiveness scores displayed by nesting lapwing parents (*n* = 74 individuals) when exposed to stuffed nest predators with nest identity as random effect (variance = 0.3252; standard deviation = 0.5703; residual variance = 0.9341; standard deviation = 0.9665), with 95% confidence intervals.

Predictors	Estimates	95% CI	*p*
(Intercept)	1.6664	1.2343–2.0985	**<0.001**
Distance to closest forest patch	−0.0018	−0.0031 to −0.0005	**0.019**
Area of closest forest patch	−0.0009	−0.0023 to 0.0006	0.286
Dummy: crow	2.0726	1.5333–2.5954	**<0.001**
Dummy: polecat	1.5790	1.0886–2.0686	**<0.001**
Forest distance: crow	0.0010	−0.0008 to 0.0028	0.305
Forest distance: polecat	0.0024	0.0008–0.0039	**0.005**
Forest area: crow	0.0009	−0.0009 to 0.0026	0.336
Forest area: polecat	0.0007	−0.0009 to 0.0024	0.401

*Note*: Bold values highlight significance levels: *≤0.05.

## 4 DISCUSSION

Our study provided a detailed investigation of nest predation risk and variability in anti‐predatory behaviours of ground‐nesting birds in a mosaic agricultural landscape, with regards to forest distance and area. We showed that predation risk on experimental nests increased with forest proximity and area of the closest forest patch. However, predation rates on lapwing nests were lower and not affected by forest characteristics, suggesting that adults could successfully protect their clutch. Parental aggressiveness appeared stronger at closer distances from forest patches. Both forest distance and forest area influenced the specific behavioural responses of breeding birds, which differed towards a mammalian nest predator.

### 4.1 Predation risk associated with forest patches

The risk of predation on experimental ground nests increased closer to forest patches. This pattern is consistent with previous studies that found higher predation rates closer to forest edges (Burger et al., 1994; Kaasiku et al., 2022; Laux et al., 2024), and generally closer to habitat edges (Batáry & Báldi, 2004; Hartley & Hunter, 1998). This pattern could be explained by higher densities of carnivorous mammals close to forests (Červinka et al., 2011; Šálek et al., 2014; Svobodová et al., 2011), and avian predators using trees as perches (Andersson et al., 2009; Berg et al., 1992). We did not record the predator species involved in our experimental design, but the local community of ground‐nest predators mainly included avian predators (Baraldi, 2025).

Forest size positively influenced predation risk. Larger patches could theoretically harbour higher densities or diversity of predators. Additionally, larger patches in the landscape could be associated with a smaller area of open habitats, which could reduce the distance between nests and habitat edges in general. While some studies have found a positive correlation between woodland surface and predator activity and density (Andren, 1992; Douglas et al., 2014; Laux et al., 2022), most empirical evidence contradicts this link (Andren, 1992; Gardiner et al., 2018; Garmendia et al., 2013). Thus, the negative effect of larger forests might be driven by site‐specific conditions and local predator communities.

Predation rates on experimental nests were four times higher than on lapwing nests. This result was expected, as both methodologies do not quantify the same environmental pressures, and both are often poorly correlated (Jayanth, 2024). The lower predation rates measured on lapwing nests indicate that populations have adapted to local interactions (Freeman et al., 2020). Moreover, regular visitation and the lack of protection for experimental nests could affect predator detection and increase predation compared with lapwing nests.

Unlike for experimental nests, the predation rate for lapwing nests did not change with forest distance and size. Some previous surveys also found no effect of forest distance on predation of lapwing nests (Bertholdt et al., 2017; Tilgar et al., 2024), while others detected a negative effect (Berg et al., 1992; MacDonald & Bolton, 2008). The difference between predation rates measured on experimental nests and lapwing nests indicates that parental defence behaviours were efficient at driving away predators (Freeman et al., 2020). Thus, parents were able to compensate for the higher predation risk related to forest patches.

Additionally, lapwings are known to nest in loose colonies, less than 100 m apart from conspecifics (Šálek & Šmilauer, 2002; Jayanth, 2024), and the effect of colony presence and size has been shown to reduce the rate of predation (Berg et al., 1992; Seymour et al., 2003). Neighbouring pairs might have further reduced the overall predation rate of lapwing nests, and the negative effect of forest proximity. However, we were not able to evaluate if the lapwing breeding density varied with forest distance in our area. Another strategy to mitigate the effect of predation could be to nest further from forest edges, as has been observed in many shorebirds (Bertholdt et al., 2017; Pálsdóttir et al., 2022). Different studies found that the negative edge effect affects nest survival within a range between 50 m (Paton, 1994), up to 4.1 km from high predation risk habitats (Storch et al., 2005). In our study area, lapwings nested between 46 and 1595 m (mean = 518 m) from forests, but still compensated for the predation risk we measured on experimental nests. Hence, the capacity of ground‐nesting birds to compensate for edge‐associated predation may vary between locations.

We observed differences in predation risk between habitats, both for experimental and lapwing nests. Differences in predation rates between habitats or agricultural schemes have been found in many studies for experimental and real nests (Bravo et al., 2023; Tilgar et al., 2024). There is evidence that vegetation cover or local prey availability influences nest predation (Ponce et al., 2018; Vander Haegen et al., 2002). In our study, predation rates were higher in fields without recent agricultural activities and with good cover. Thus, differences between habitats were probably influenced by the availability of alternative prey, including rodents or arthropods, attracting opportunistic predators like raptors and corvids (Jayanth, 2024).

### 4.2 Aggressive responses associated with forest patches

When exposed to nest predator dummies, aggressiveness scores increased with forest proximity. As stated in our hypotheses, this suggested that individuals acted more aggressively closer to the forest. In another study, geese exhibited shorter flushing distances in areas with higher predator densities, suggesting local behavioural adaptations to predation (Clermont et al., 2021). Several passerine species acted more aggressively towards experimental predators in locations where the given predators occurred (Liu et al., 2025; Sandoval & Wilson, 2012). In our case, lapwings displayed aggressive behaviours where predation pressure was also higher. Ultimately, these combined results suggest that birds show specific responses adapted to the local risk of predation.

Contrary to our prediction, we did not find an effect of forest area on the aggressiveness score. As larger forest patches have not been related to higher predator densities (Červinka et al., 2011), this could explain why forest size might not influence behavioural traits in our study.

Incubation stage did not affect aggressiveness in our study. Similar dummy experiments have also failed to detect an increase in aggressiveness with nest age (Brynychová et al., 2022; Kis et al., 2000). The lack of effects for interaction terms between dummy and both year and presentation order indicated no habituation and no effect of changing the design between years.

### 4.3 Specific aggressive responses towards predatory guilds

Aggressiveness level did vary between stimuli, with higher scores recorded against the two predators compared with the wood log. As large predators are easily detected, responses to harmful dummies have been generally higher in other studies (Brynychová et al., 2022; Elliot, 1985), in line with our findings. These observations supported our hypotheses, which suggested different reactions to predatory guilds.

Interaction term between forest distance and crow did not differ from interaction with the log. This result suggested a tendency of birds to act more aggressively closer to the forest when facing either the crow or the log. In other studies, the link between corvid abundance and the distance to forest patches varied strongly between locations: sometimes increasing with distance to trees (Berg et al., 1992), otherwise showing no correlation (Douglas et al., 2014). Plausibly, crows and other avian predators might be encountered regularly closer to trees, which are used as perches (Andersson et al., 2009). Thus, lapwings' nests close to forest patches might be more easily detected and predated by corvids, and parents may behave aggressively accordingly. It remains unclear why lapwings behaved more aggressively towards the wood log closer to forests. Since it was placed vertically next to the nest, lapwings might have initially identified the log as a threatening object.

Lapwings behaved differently towards the stuffed polecat with regard to forest distance, as indicated by the significant interaction. However, contrary to our prediction, lapwings acted more aggressively towards the mustelid when further from forest patches. The abundance of mammalian predators is higher at closer distances to forest edges (Červinka et al., 2011; Kaasiku et al., 2022). Thus, nests at closer distances were more likely to have encountered mammals before. However, mammals mostly hunt at night (Seymour et al., 2003; Teunissen et al., 2008), and the polecat presented during daytime might appear abnormal to the birds. Among shorebirds, attacks are rarely displayed against mammalian predators, for which ground displays are preferred (Byrkjedal, 1987; Elliot, 1985), here associated with lower aggressiveness scores. Ultimately, birds around forest edges could have acted more cautiously as mustelids can prey on adult birds (Rysavá‐Nováková & Koubek, 2009). When exposed to harmful mammalian predators, aggressive birds maintained a distance and avoided contact (Brynychová et al., 2022; Elliot, 1985), or estimated the risk through predator inspection before engaging in attacks (Carlson & Griesser, 2022).

### 4.4 Future directions

Our study showed that higher local predation pressure was compensated for and linked to specific behavioural responses of ground‐nesting birds. Despite extensive work on edge effects, few studies have incorporated a behavioural component. This lack of knowledge limits our understanding of how different populations might be affected by habitat change and fragmentation. While our study highlights that birds were able to compensate for negative edge effects, other studies have not reported the same. These conflicting results suggest a strong effect of site‐specific variables, like local predator communities. Species traits are also likely to influence predation rates and behavioural responses. Theoretically, species nesting under vegetation cover or raising altricial hatchlings might reduce defensive behaviours so as not to attract attention to the nests. Long‐lived species or populations in tropical regions might also invest more in survival or later breeding attempts and avoid investing energy in risky behaviours. To fully understand which parameters can lead to variable responses of populations to edge effects, a comparative survey should be performed across various species, habitats and locations.

Previous studies have linked nest site characteristics with the prior experience of breeding birds at the individual level, but we lack evidence on how individuals are distributed in the landscape and which cues are used to select nest sites. With the development of tracking technology, monitoring individuals over several breeding attempts could help to evaluate the repeatability of nesting decisions at very fine scales. Long‐term surveys would also help to correlate nesting characteristics with temporal variations in predation that birds are experiencing. Individually marking birds would also inform which specific adults invest in defence and highlight the roles of each sex and the involvement of conspecifics in the colony.

Additional focus should be given to the proximate causes responsible for variation in behavioural traits within the spatial framework, as our study did not allow us to identify the underlying processes. Higher aggressiveness scores close to forest edges could be the result of behavioural flexibility displayed by individuals, implying that birds might display flexibility in nest site selection and low repeatability of aggressiveness traits. Conversely, positive selection of aggressive individuals close to areas with high predation risk would lead to a specific distribution of aggressive individuals and low flexibility in nest site selection.

Possible future investigations could strengthen the concept of the landscape‐of‐fear (Gaynor et al., 2019). Moreover, accounting for landscape composition in combination with species behaviour and adaptation will be essential for future conservation programmes.

## 5 CONCLUSION

Our study showed that predation risk for ground nests increased with the presence of adjacent forests in a mosaic landscape. However, this risk was counterbalanced by breeding birds, and lapwing nest predation rates were not affected by forest proximity. Birds that established their territories at different distances from forest patches showed variable levels of aggressiveness towards potential nest predators associated with forest habitats. Breeding birds appeared more aggressive towards avian predators closer to forests, but were more aggressive towards mammals at greater distances. These results highlight the importance of behavioural differences between individuals within heterogeneous landscapes.

## AUTHOR CONTRIBUTIONS

Guillaume Dillenseger and Petr Veselý led the conception of the study and designed the methodology, supported by Justine Delautre and Vojtěch Kubelka; Justine Delautre led data collection during 1 year, Zora Marchal, Ankitha Jayanth, and Guillaume Dillenseger during the other, supported by Vojtěch Kubelka; Ankitha Jayanth, Guillaume Dillenseger, and Justine Delautre equally analysed the data; Guillaume Dillenseger led the writing of the manuscript. All authors contributed critically to the drafts and gave final approval for publication. Justine Delautre realised the additional drawings within the figures.

## CONFLICT OF INTEREST STATEMENT

Authors declared having no conflict of interest.

## STATEMENT ON INCLUSION

Our study brings together authors from different countries, at different academic levels, with local researchers involved in the country the study was carried out. All authors took significant parts in the realisation of the study, combining personal skills and various perspectives. A specific effort was given to reference previous work carried out in the region, although no publication in Czech language was ultimately cited.

## Supporting information


**Data S1.** Ethogram of behaviours displayed by breeding lapwings of 16 nests, during 2024–2025 breeding seasons, during 42 dummy presentation experiments.

## Data Availability

Data available from Figshare repository: https://doi.org/10.6084/m9.figshare.29877485 (Dillenseger, 2026).
