# Supplementary material for: Predator‐guild‐specific parental responses mitigate higher predation risk on ground nests close to forest patches in a mosaic landscape
Source: J Anim Ecol. 2026 May 18;95(7):1151–62. doi: 10.1111/1365-2656.70278 (PMC13322176; doi:10.1111/1365-2656.70278)
Supplement: Supplementary file 1 — Data S1. Ethogram of behaviours displayed by breeding lapwings of 16 nests, during 2024–2025 breeding seasons, during 42 dummy presentation experiments. [file JANE-95-1151-s001.docx]

**Higher nest predation risk close to forests is compensated by parental responses towards avian and mammalian predators in a mosaic landscape**

Supporting Information

**Supporting Information S1.** Ethogram of behaviours displayed by breeding lapwings of 16 nests, during 2024–2025 breeding seasons, during 42 dummy presentation experiments.

| Behaviours | Description |
| --- | --- |
| Away | The individual is staying at an unknown distance, and is not detected by the observer on the field |
| Ignoring | The individual is performing any behaviour that does not fit under the other defined categories, and does not appear directed towards the dummy (e.g., preening, interacting with conspecifics, heterospecifics or experimenter) |
| Incubating | The individual is sitting on its nest |
| Flying | The individual is flying across the environment, not directly above or around the dummy, and does not appear directed towards the dummy |
| Vigilance far | The individual is standing or walking on the ground, possibly interspersed with pecking motions, with visibly extended neck that suggests an alert state, at distance >5 m from the dummy |
| Vigilance close | The individual is standing or walking on the ground, possibly interspersed with pecking motions, with visibly extended neck that suggests an alert state, at distance ≤5 m from the dummy |
| Broken-wing display | The individual is standing or walking on the ground with one wing partially deployed, possibly dragging it and doing irregular beating movements, acting as injured |
| Circling | The individual is flying directly above or around the dummy without approaching it, with or without vocalisations |
| Attacking | The individual is flying above or around the dummy and is constantly diving towards parabolic movements withing ≤3 m towards it, with or without vocalisations. If the individual is not directly doing an attack after another, it is considered “Circling” in between |
